# Exploratory Laparotomy and Gastrotomy for Long Metallic Gastric Foreign Bodies Presenting With Hematemesis: A Case Report

**DOI:** 10.7759/cureus.109311

**Published:** 2026-05-20

**Authors:** Hugo E Mora Moreno, Noel García Gonzalez, Marisela Silva García, Cesar A Durán Ramírez, Omar J Madrigal Flores

**Affiliations:** 1 General Surgery, Hospital General Dr. Miguel Silva, Morelia, MEX

**Keywords:** foreign bodies, gastrointestinal hemorrhage, laparotomy, self-injurious behavior, stomach

## Abstract

Foreign body ingestion in adults is a clinically significant condition that may lead to serious complications, particularly in cases involving intentional ingestion of high-risk objects. We report the case of a 34-year-old woman who presented with a two-day history of hematemesis after intentionally ingesting two metallic foreign bodies approximately two weeks before admission. Imaging studies, including abdominal radiography and computed tomography, revealed elongated metallic objects measuring up to 6.8 cm within the stomach, associated with focal gastric wall thickening. Given the size of the objects, the presence of gastrointestinal bleeding, and the unavailability of therapeutic endoscopy, surgical management was pursued. The patient underwent exploratory laparotomy with gastrotomy, allowing the successful extraction of the foreign bodies and primary closure reinforced with an omental patch. Her postoperative course was uneventful, with progressive recovery and no recurrence of symptoms at follow-up. This case underscores the importance of early recognition, appropriate imaging, and timely intervention in high-risk foreign body ingestion, as well as the role of surgical management when endoscopic treatment is not feasible.

## Introduction

Foreign body ingestion is a common clinical problem encountered in emergency departments and surgical practice, with presentations ranging from asymptomatic cases to severe, life-threatening complications. Although most ingested objects pass spontaneously through the gastrointestinal tract, approximately 10-20% require endoscopic intervention, and a smaller proportion require surgical management because of complications such as obstruction, perforation, or gastrointestinal bleeding [1,2].

In adults, foreign body ingestion is frequently associated with high-risk groups, including patients with psychiatric disorders, intentional ingestion, substance use, or incarceration. In contrast to accidental ingestion in children, intentional ingestion in adults more commonly involves multiple, sharp, or elongated objects, increasing the risk of mucosal injury, impaction, perforation, and the need for invasive intervention [2-4]. Recent epidemiological data also suggest a rising trend in adult cases requiring endoscopic or surgical management, highlighting the importance of timely diagnosis and appropriate therapeutic planning [3].

Management depends primarily on the characteristics of the ingested object, particularly its size, shape, composition, and location within the gastrointestinal tract. Objects longer than 5-6 cm are less likely to pass beyond the pylorus and are associated with an increased risk of impaction, mucosal injury, and subsequent complications [1,2]. Imaging plays a central role in evaluation, with plain radiography and computed tomography (CT) helping determine the number, location, size, and potential complications of retained foreign bodies [5].

Gastrointestinal bleeding is an uncommon but clinically relevant presentation of foreign body ingestion and may indicate mucosal erosion, ulceration, or direct injury caused by the retained object. In these cases, early endoscopic removal is recommended when feasible. However, surgical intervention may be required when endoscopic management is unavailable, unsuccessful, or contraindicated, particularly in patients with large, sharp, elongated, or otherwise high-risk objects [1,2].

Herein, we present a case of intentional ingestion of long metallic foreign bodies presenting with hematemesis, requiring surgical retrieval because therapeutic endoscopy was unavailable at the time of presentation. This case highlights the importance of early recognition, appropriate imaging, and individualized management in high-risk foreign body ingestion.

## Case presentation

A 34-year-old woman presented to the emergency department with a two-day history of hematemesis. She reported intentionally ingesting two metallic foreign bodies approximately two weeks before admission during a suicide attempt. She had no documented history of prior suicide attempts or previous psychiatric treatment. At that time, she remained asymptomatic and did not seek medical care.

Two days before presentation, she developed two episodes of hematemesis without an identifiable precipitating factor, accompanied by progressively worsening dull, aching epigastric pain. She denied fever, nausea, additional non-bloody vomiting, diarrhea, melena, hematochezia, or changes in bowel habits.

Her surgical history was notable for three prior cesarean sections, the most recent performed 10 years earlier.

On physical examination, the patient was alert and oriented. Her vital signs were stable, with a blood pressure of 118/68 mmHg, a heart rate of 74 beats/min, a respiratory rate of 18 breaths/min, a temperature of 36.3°C, and an oxygen saturation of 96% on room air. Abdominal examination revealed a flat abdomen with preserved bowel sounds. Localized tenderness was present in the epigastrium, without guarding, rebound tenderness, or other signs of peritoneal irritation.

Initial laboratory studies were largely within normal limits, except for mild leukocytosis and a mild elevation in alanine aminotransferase to 66 U/L. The white blood cell count was 10.1×10³/µL, and the hemoglobin level was 13.8 g/dL, with no laboratory evidence of significant acute blood loss (Table 1). 

**Table 1:** Initial laboratory findings at admission Initial laboratory values were largely within normal limits, except for mild leukocytosis and mildly elevated alanine aminotransferase. Hemoglobin was preserved, with no laboratory evidence of significant acute blood loss.

Parameter	Result	Reference range
White blood cell count	10.1×10³/µL	4.5-10.0×10³/µL
Hemoglobin	13.8 g/dL	12.0-15.5 g/dL
Platelet count	315×10³/µL	150-400×10³/µL
Lactate dehydrogenase	154 U/L	120-300 U/L
C-reactive protein	0.2 mg/L	0-6 mg/L
Aspartate aminotransferase	27 U/L	0-40 U/L
Alanine aminotransferase	66 U/L	0-41 U/L
Glucose	78 mg/dL	74-109 mg/dL

A plain abdominal radiograph demonstrated two linear radiopaque foreign bodies projecting over the upper abdomen, in the expected region of the distal esophagus and stomach (Figure 1).

**Figure 1:**
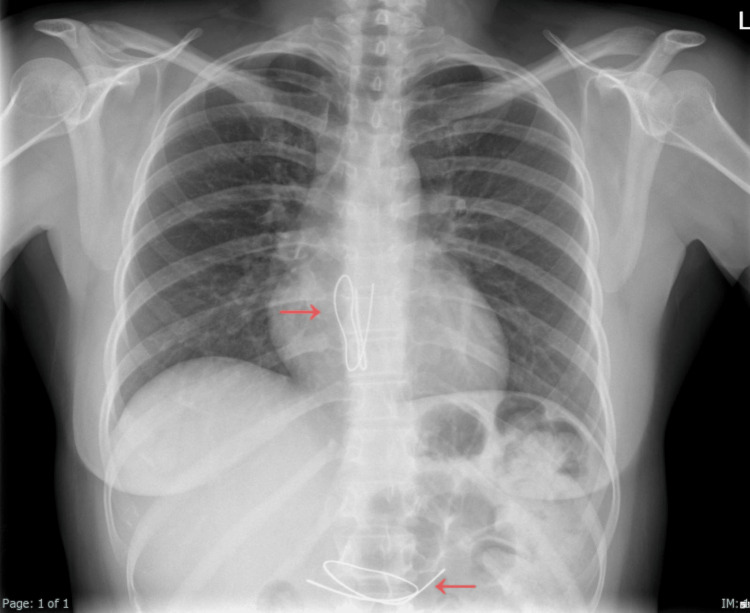
Plain abdominal radiograph showing radiopaque metallic foreign bodies Anteroposterior abdominal radiograph demonstrating two elongated radiopaque foreign bodies projected over the upper abdomen, corresponding to the expected gastric region. The arrows indicate the metallic foreign bodies.

To further characterize these findings, a non-contrast CT scan of the abdomen was obtained. CT confirmed the presence of metallic-density foreign bodies within the stomach, the largest measuring approximately 6.8 cm in length (Figures 2-3).

**Figure 2:**
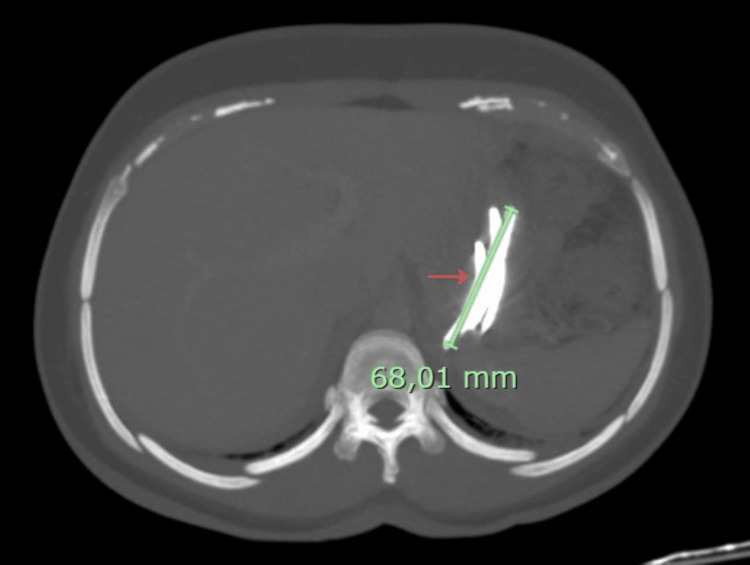
Axial computed tomography showing an intragastric metallic foreign body Non-contrast axial computed tomography image demonstrating a hyperdense metallic foreign body within the stomach. The measured segment is approximately 6.8 cm in length, and the arrow indicates the retained foreign body.

**Figure 3:**
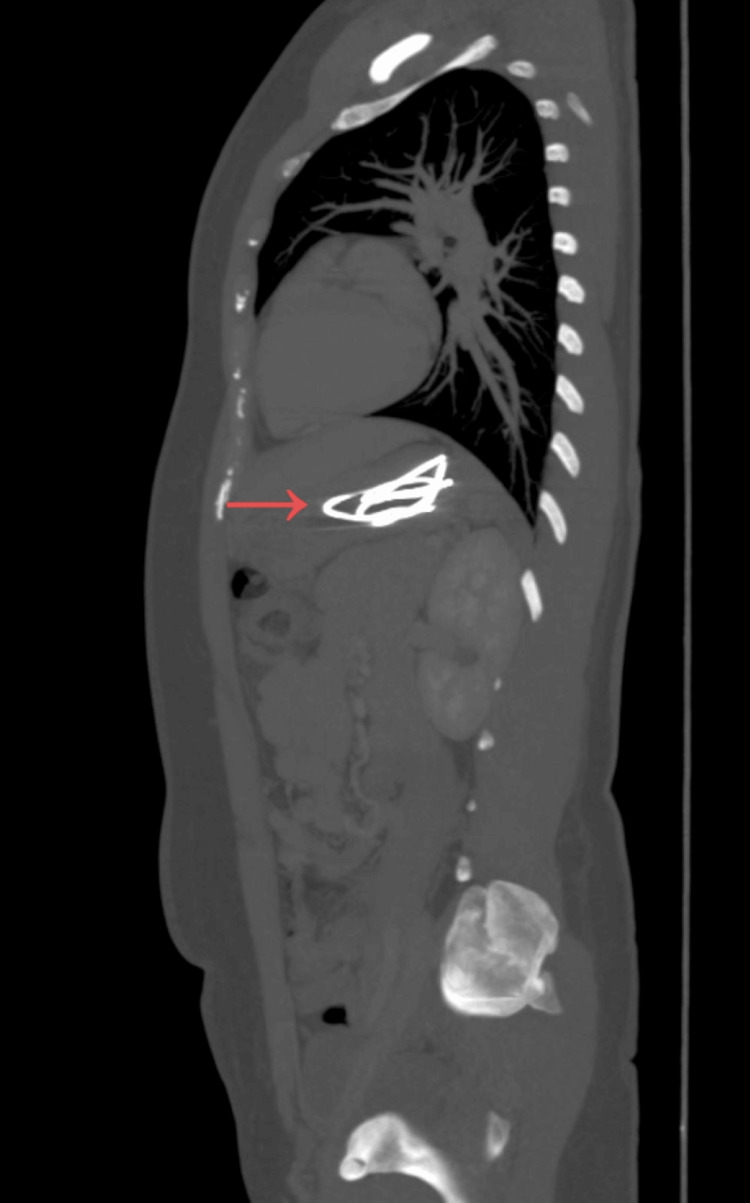
Sagittal computed tomography reconstruction showing elongated metallic foreign bodies within the stomach Sagittal computed tomography reconstruction demonstrating elongated metallic foreign bodies within the gastric lumen. The arrow highlights the intragastric location of the retained metallic objects.

These findings were associated with focal gastric wall thickening measuring up to approximately 1.2 cm, suggestive of localized inflammatory changes (Figure 4). No free intraperitoneal air, perforation, or distal bowel obstruction was identified. 

**Figure 4:**
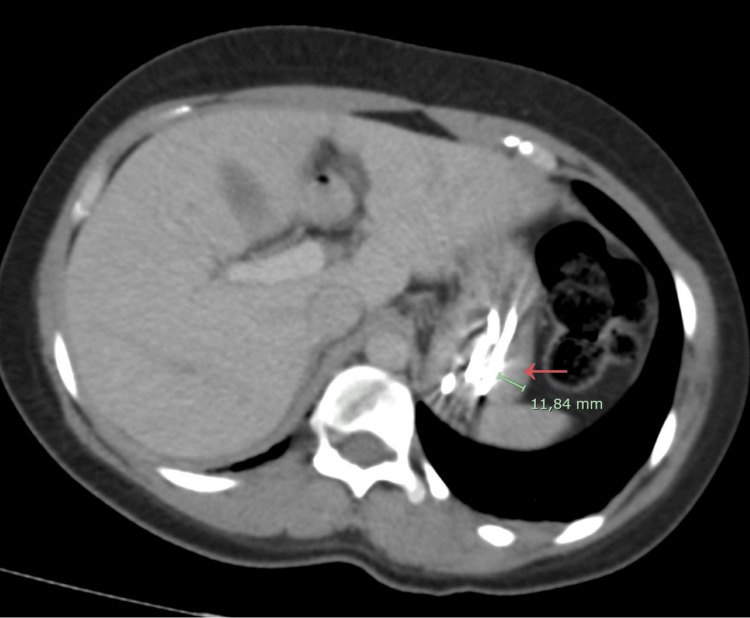
Focal gastric wall thickening adjacent to the retained metallic foreign body Axial computed tomography image showing focal gastric wall thickening adjacent to the retained metallic foreign body, measuring approximately 11.8 mm. The arrow indicates the area of localized wall thickening, suggestive of an inflammatory reaction.

Given the size, elongated morphology, and intragastric location of the foreign bodies, together with the presence of upper gastrointestinal bleeding, endoscopic retrieval was initially considered. However, because therapeutic endoscopy was not available at our institution at the time of presentation, surgical management was performed.

The patient underwent exploratory laparotomy. During surgery, a firm intragastric foreign body was palpated. A 2 cm anterior gastrotomy was created in the gastric body, allowing the direct visualization and removal of two metallic foreign bodies (Figures 5-6). A nasogastric tube was inserted intraoperatively for gastric decompression. The gastrotomy was closed primarily in two layers using absorbable sutures; the inner layer was closed with a continuous absorbable suture, and the outer seromuscular layer was reinforced with interrupted absorbable sutures. The repair was reinforced with an omental patch. A closed-suction drain was placed adjacent to the repair site. The nasogastric tube was removed on postoperative day 4.

**Figure 5:**
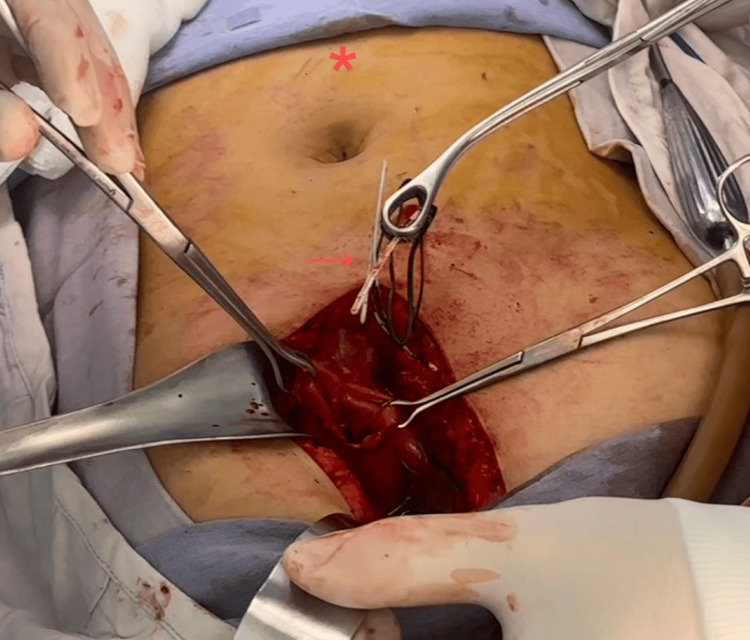
Intraoperative retrieval of metallic foreign bodies through anterior gastrotomy Intraoperative photograph showing the extraction of the metallic foreign bodies through an anterior gastrotomy in the gastric body via a supraumbilical laparotomy incision. The arrow indicates the retrieved metallic object during surgical removal. The asterisk marks the caudal/foot end of the patient to clarify the orientation of the image; the opposite side corresponds to the cephalad/head end.

**Figure 6:**
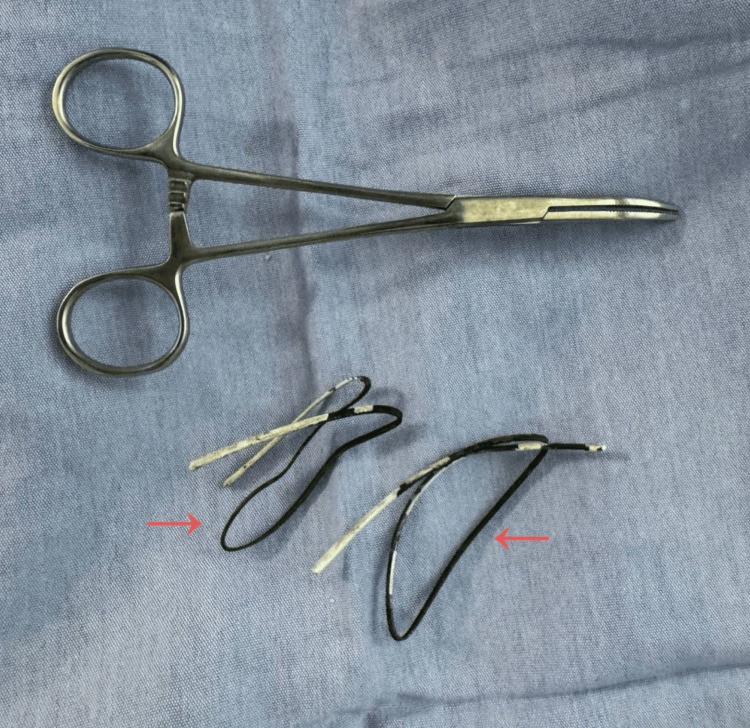
Gross specimen of the extracted metallic foreign bodies Gross specimen photograph showing the two elongated metallic foreign bodies retrieved from the stomach during surgical exploration. A surgical clamp is shown for size reference, and the arrows indicate the extracted foreign bodies.

Postoperatively, the patient received intravenous ceftriaxone and metronidazole, proton pump inhibitor therapy, and analgesia. She was kept without oral intake for 48 hours. Her postoperative course was uneventful. She remained hemodynamically stable, with minimal serous drain output and no clinical signs of infection, recurrent bleeding, or leakage.

Oral intake was gradually resumed on postoperative day 3 and was well tolerated. Bowel function returned appropriately, and no further episodes of hematemesis were observed. Given the intentional nature of the ingestion, psychiatric evaluation was requested during hospitalization, and outpatient mental health follow-up was recommended after discharge. The drain was removed before discharge, and the patient was discharged on postoperative day 7 with outpatient follow-up.

At the one-month follow-up, she reported adequate oral intake, normal bowel movements, and no recurrence of hematemesis. Physical examination showed a soft, non-tender abdomen without signs of peritoneal irritation. The skin sutures were removed without complications.

## Discussion

Foreign body ingestion in adults is a clinically heterogeneous condition, with management determined by the characteristics of the ingested object, its anatomical location, the timing of presentation, and the presence or absence of complications. Although most ingested foreign bodies pass spontaneously through the gastrointestinal tract, a clinically relevant proportion requires endoscopic intervention or surgery, particularly when complications such as obstruction, perforation, or gastrointestinal bleeding are present [6].

Endoscopic management has become central in the treatment of upper gastrointestinal foreign bodies, especially when the object is accessible and has high-risk features. Large adult series have shown that emergency endoscopic removal can be safe and effective when performed according to established recommendations and when adequate expertise and equipment are available [7]. Recent epidemiological data also suggest that foreign body ingestion remains an important and evolving problem in adult populations, with increasing attention to cases requiring endoscopic or surgical management [8].

Intentional ingestion, as observed in our patient, is commonly associated with psychiatric illness or self-injurious behavior and may involve multiple, sharp, elongated, or otherwise high-risk objects. These characteristics increase the risk of mucosal injury, impaction, perforation, and the need for invasive management [9]. Delayed presentation is also clinically relevant, as prolonged contact between a retained foreign body and the gastrointestinal wall may increase the likelihood of pressure-related mucosal injury, bleeding, or perforation. In the present case, the patient remained asymptomatic for approximately two weeks after ingestion before developing hematemesis and progressive epigastric pain.

Object size and morphology are critical determinants of management. Long, sharp, or pointed foreign bodies are associated with an increased risk of impaction, mucosal injury, bleeding, and perforation; objects longer than 5-6 cm are less likely to pass through the pylorus and duodenal sweep [10]. In our case, the largest metallic foreign body measured approximately 6.8 cm, placing it within a high-risk category. The presence of hematemesis further suggested mucosal injury, likely related to prolonged contact between the retained metallic objects and the gastric wall. This was supported by CT findings showing focal gastric wall thickening adjacent to the foreign body.

Endoscopic retrieval is generally considered the preferred first-line approach for accessible upper gastrointestinal foreign bodies when feasible, particularly for sharp, elongated, or otherwise high-risk objects. Early endoscopic management allows removal before progression to perforation, obstruction, or deeper mural injury [11]. However, the success and availability of endoscopic treatment depend on several factors, including object size, morphology, location, timing of presentation, local expertise, and institutional resources.

In the present case, endoscopic retrieval was initially considered because the foreign bodies were located in the stomach and the patient had evidence of upper gastrointestinal bleeding. However, therapeutic endoscopy was not available at our institution at the time of presentation. In this context, surgical retrieval was considered the most appropriate definitive management strategy, given the elongated metallic nature of the foreign bodies, their size, the delayed presentation, and the presence of hematemesis. Clinical evidence supports individualized decision-making, as intervention may not be necessary in all cases but becomes appropriate when high-risk features, symptoms, or complications are present [12].

Exploratory laparotomy with anterior gastrotomy allowed the definitive removal of the two metallic foreign bodies and the direct assessment of the stomach. Although less invasive approaches are preferred when available, open surgical management remains a safe and effective alternative in selected patients, especially when endoscopic treatment cannot be performed or when complications are suspected. Timely intervention is important to prevent progression to more severe complications, including perforation, abscess formation, peritonitis, persistent bleeding, or distal obstruction [13].

Gastrointestinal bleeding is an uncommon but clinically significant presentation of foreign body ingestion and may indicate mucosal erosion, ulceration, or direct mechanical injury caused by the retained object. In this case, hematemesis likely reflected gastric mucosal irritation or ulceration caused by the retained metallic objects. Importantly, bleeding occurred in the absence of radiological evidence of perforation, emphasizing that clinically relevant mucosal injury may occur even when free air or generalized peritonitis is absent [6,13].

This case highlights several important considerations. First, intentional ingestion of long metallic objects should be considered a high-risk scenario, even when the patient is initially asymptomatic [9]. Second, although endoscopic retrieval remains the preferred first-line approach for accessible upper gastrointestinal foreign bodies, surgery remains essential when endoscopy is unavailable, unsuccessful, or not feasible [7,11,12]. Third, hematemesis should raise concern for underlying mucosal injury and prompt expedited diagnostic and therapeutic management [6,13]. Finally, because intentional ingestion may reflect underlying psychiatric disease or suicidal behavior, psychiatric evaluation and appropriate mental health follow-up are important components of comprehensive care [9].

This report is limited by its single-patient design and by the absence of endoscopic evaluation, which was unavailable at the time of presentation. Nevertheless, it illustrates the importance of individualized decision-making and reinforces the role of surgical management when therapeutic endoscopy cannot be performed in the setting of high-risk foreign body ingestion.

## Conclusions

Intentional ingestion of long metallic foreign bodies is a high-risk clinical presentation that may lead to significant complications, including mucosal injury, impaction, perforation, and upper gastrointestinal bleeding. Early recognition is essential, particularly in patients with psychiatric risk factors or delayed presentation, as symptoms may develop after an initially asymptomatic period. Appropriate imaging, including plain radiography and CT, plays a central role in identifying the number, size, location, and potential complications of retained foreign bodies. While endoscopic retrieval remains the preferred first-line approach for accessible upper gastrointestinal foreign bodies, surgical removal is a safe and effective alternative when endoscopy is unavailable, unsuccessful, or not feasible. This case emphasizes the importance of timely intervention and individualized management based on the patient's clinical status, foreign body characteristics, complication risk, and available institutional resources.
